# Changes in surface water drive the movements of Shoebills

**DOI:** 10.1038/s41598-021-95093-5

**Published:** 2021-08-04

**Authors:** Marta Acácio, Ralf H. E. Mullers, Aldina M. A. Franco, Frank J. Willems, Arjun Amar

**Affiliations:** 1 School of Environmental Sciences, University of East Anglia, Norwich, NR4 7TJ UK; 2 Animal Management, Van Hall Larenstein, University of Applied Sciences, Agora 1, 8934 CJ Leeuwarden, The Netherlands; 3 FitzPatrick Institute of African Ornithology, DSI-NRF Centre of Excellence, University of Cape Town, Rondebosch, Cape Town, 7701 South Africa; 4 Kigelia Solutions, Chisamba Park, Box 12, Fringilla, Zambia; 5 BirdWatch Zambia, 25 Joseph Mwilwa Rd, Lusaka, Zambia

**Keywords:** Wetlands ecology, Tropical ecology, Ecology, Animal migration

## Abstract

Animal movement is mainly determined by spatial and temporal changes in resource availability. For wetland specialists, the seasonal availability of surface water may be a major determinant of their movement patterns. This study is the first to examine the movements of Shoebills (*Balaeniceps rex*), an iconic and vulnerable bird species. Using GPS transmitters deployed on six immature and one adult Shoebills over a 5-year period, during which four immatures matured into adults, we analyse their home ranges and distances moved in the Bangweulu Wetlands, Zambia. We relate their movements at the start of the rainy season (October to December) to changes in Normalized Difference Water Index (NDWI), a proxy for surface water. We show that Shoebills stay in the Bangweulu Wetlands all year round, moving less than 3 km per day on 81% of days. However, average annual home ranges were large, with high individual variability, but were similar between age classes. Immature and adult Shoebills responded differently to changes in surface water; sites that adults abandoned became drier, while sites abandoned by immatures became wetter. However, there were no differences in NDWI of areas used by Shoebills before abandonment and newly selected sites, suggesting that Shoebills select areas with similar surface water. We hypothesise that the different responses to changes in surface water by immature and adult Shoebills are related to age-specific optimal foraging conditions and fishing techniques. Our study highlights the need to understand the movements of Shoebills throughout their life cycle to design successful conservation actions for this emblematic, yet poorly known, species.

## Introduction

One of the key challenges in ecology is to understand how environmental fluctuations drive animal movements. Changes in the environment can alter resource distribution, which consequently determines animal migratory^1–3^ and, local, movements^4,5^. In wetlands, the distribution of surface water is one of the main determinants of species’ spatial distribution^6–8^ and individual movements^9,10^. In tropical systems with strongly seasonal environments, prolonged periods of drought followed by extreme floods can lead to striking changes in habitat suitability^9,11^ and drive the large-scale movements of waterfowl^12^, due to fluctuations in the abundance and availability of foraging resources^13^.

The way individuals explore the environment can change as they age^14,15^, and recent advances in GPS tracking technology and increases in device longevity, have enabled the detailed study of individual movements for several years or even throughout lifetimes. This has unravelled differences between adults and juveniles in space use^15,16^, habitat selection^14,17^, and timing^18–20^ and efficiency of movements^20–22^. Understanding the drivers of movement of long-lived birds relies on information on the spatial and temporal dynamics of movement at different ages in relation to environmental variables. Such information has only been available relatively recently, through the integration of data from GPS trackers with remotely sensed environmental data^23–25^. Indices based on satellite imagery have been increasingly used to interpret environmental conditions and infer ecological processes^23,25^. The Normalized Difference Water Index (NDWI) proposed by McFeeters^26^ is an index that uses remotely sensed imagery to map surface water. The NDWI delineates and highlights open water by distinguishing it from vegetation and bare soil, and has mostly been used to map waterscapes in urban settings^27,28^. More recently, this index has been used to map surface water for animal movement studies^12^, and to identify suitable habitat and inform area protection for shorebird species^29^.

The Shoebill (*Balaeniceps rex*) is an iconic wetland specialist, with a patchy distribution in central-eastern Africa, from South Sudan to Zambia^30,31^. The Shoebill is a large long-lived species, categorised as *Vulnerable* by the IUCN. Shoebills have a declining population trend, due to habitat degradation and loss, illegal bird trade and disturbance by humans^31,32^. The global population estimate for the species is 5000–8000 individuals, although large uncertainty around this estimate exists, given that this species is cryptic and found in inaccessible areas^31^. Shoebills inhabit permanent swampy wetlands with seasonal flooded grasslands, where they prey on fish in shallow waters or use floating vegetation as fishing sites^33,34^. Despite being a highly emblematic species, there are very few studies on Shoebill ecology, and existing studies have focused on deriving local population estimates^30,35^, and better understanding their foraging^33,34^ and breeding ecology^36,37^. This species is believed to be sedentary, staying in the same region all year long^32,38^; however, to date, the movement ecology of the Shoebill is completely unknown, which is unsurprising given that very few birds have ever been ringed and no previous tracking studies have occurred on this species. Being a species of high conservation concern, as well as an important source of tourism revenue^39^, it is critical to improve our knowledge of Shoebill ecology and habitat requirements to implement effective conservation measures^32^.

Using GPS tracking data collected over 5 years, we characterise the movements of immature and adult Shoebills in the Bangweulu Wetlands, Zambia. In common with many other areas occupied by Shoebills, the Bangweulu Wetlands undergoes dramatic changes in water levels between the dry (breeding season) and the wet season. We therefore hypothesise that changes in surface water drive the movements of Shoebills, and that their selected areas have similar surface water. Using the NDWI as a proxy for surface water, we compare (1) the NDWI of areas while Shoebills were present with the NDWI of the same areas the week after the birds left (to examine how these abandoned areas change), and (2) the NDWI of areas used by Shoebills the last week before abandonment with the NDWI of areas the first week after Shoebill arrival (to examine whether they select for similar habitats in relation to surface water). We explore these questions for both adult and immature birds. By analysing the movements of Shoebills in different life stages, and how these movements relate to available surface water, a key environmental factor for wetlands, our goal is to improve our ecological understanding of Shoebills, to ultimately inform the conservation of this mostly unknown and emblematic species.

## Results

We tracked 11 Shoebills in the Bangweulu Wetlands, Zambia, between December 2011 and October 2018 and collected 119,321 valid GPS positions (Table 1). We obtained 47,134 GPS positions for six Shoebills tracked as immatures and 44,985 GPS positions of five Shoebills tracked as adults. All other GPS positions were from juveniles (n = 4), which died or disappeared before they became immatures and were thus excluded from this research, also because they remained near the nest for a long period after fledging. From the adult GPS positions, 28,057 locations were from 4 immature Shoebills that matured into adults during the tracking period, and 16,928 GPS locations from the one individual tagged as a breeding adult.

**Table 1:** Information for the tracked Shoebills: age of the individual at the time of logger deployment, start and end dates of tracking, total number of valid GPS positions, excluding outliers, and total number of tracking days as immature and adult.

Bird ID	Age	Start of tracking	End of tracking	Number of GPS positions	Immature tracking days	Adult tracking days
521	Juvenile	03/12/2011	28/10/2014	10,763	617	–
514		09/09/2012	26/11/2013	2187	–	–
518		15/09/2012	21/04/2013	2537	–	–
520		03/08/2013	02/05/2018	19,335	725	623
509		26/08/2013	05/03/2018	18,998	724	634
510		30/08/2013	14/11/2013	962	–	–
515		02/09/2013	15/05/2017	16,138	728	347
512		10/09/2013	29/10/2018	21,224	724	863
516		15/11/2013	05/01/2015	3480	–	–
511		28/10/2014	08/06/2016	6769	366	–
517	Adult	29/07/2013	15/08/2017	16,928	–	1444

### Spatial analysis

**Figure 1:**
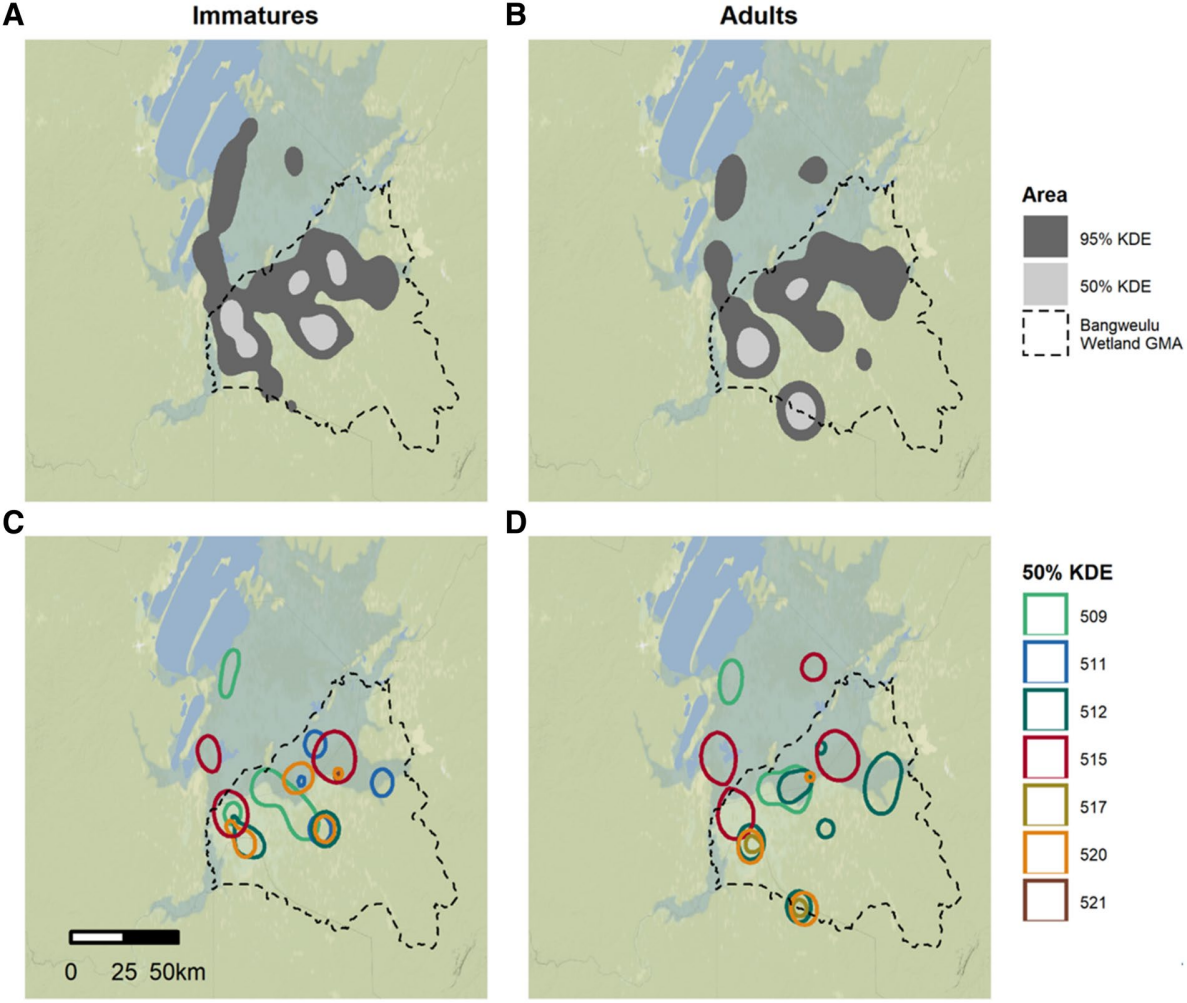
Cumulative 95% and 50% kernel density estimations for all tracked **(A)** immature and **(B)** adult Shoebills, and cumulative 50% kernel density estimation for each **(C)** immature and **(D)** adult individual, based on the GPS tracking periods indicated in Table 1. The dashed line indicates the border of the Bangweulu Wetlands Game Management Area.

Shoebill annual home range was similar in size for adults and immatures (mean 95% kernel = 1514 km^2^ (± 1172) and 1547 km^2^ (± 1296) for adults and immatures, respectively; Fig. 1, Table 2). There was large individual variation in home range size, both for immatures (range 95% kernel: 233 km^2^ and 2628 km^2^) and adults (range 95% kernel: 304 km^2^ and 3375 km^2^) (Table 2).

**Table 2:** Individual average annual home range area (in km^2^ ± standard deviation) and total average home range of immature and adult Shoebills, estimated as the 95% and 50% kernel, based on the GPS tracking periods indicated in Table 1.

ID	Immature		Adult	
	95% kernel (km^2^)	50% kernel (km^2^)	95% kernel (km^2^)	50% kernel (km^2^)
521	233 (± 318)	46 (± 63)	–	–
520	1094 (± 585)	212 (± 129)	1039 (± 866)	145 (± 124)
509	2458 (± 617)	431 (± 59)	2167 (± 241)	389 (± 85)
515	2628 (± 2309)	403 (± 359)	3375	652
512	1585 (± 360)	257 (± 56)	2167 (± 474)	343 (± 133)
511	981	200	–	–
517	–	–	304 (± 108)	54 (± 15)
All individuals	1547 (± 1296)	263 (± 204)	1514 (± 1172)	247 (± 204)

**Figure 2:**
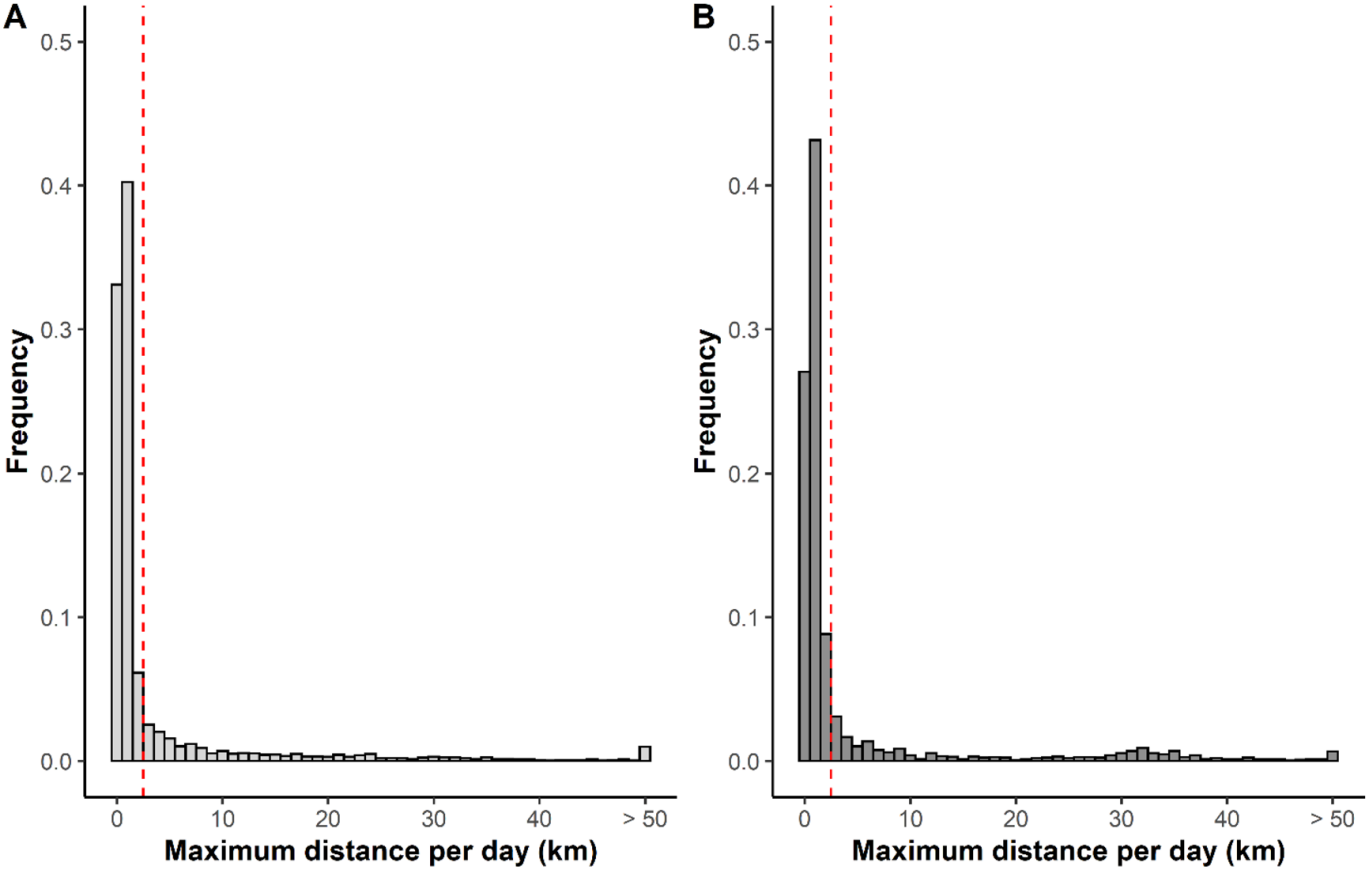
Frequency of the maximum daily distance (in Km’s) moved by **(A)** immature and **(B)** adult Shoebills. The dashed red line indicates the threshold that captures 80% of movements, used to define Shoebills’ moving days.

For both adults and immatures, the distribution of the maximum daily distance moved was highly skewed (Fig. 2). On most days both age classes moved relatively short distances (median values; adults: 0.84 km/day, immatures: 0.73 km/day). For both age classes, on 81% of days, birds moved less than 3 km (Fig. 2).

**Figure 3:**
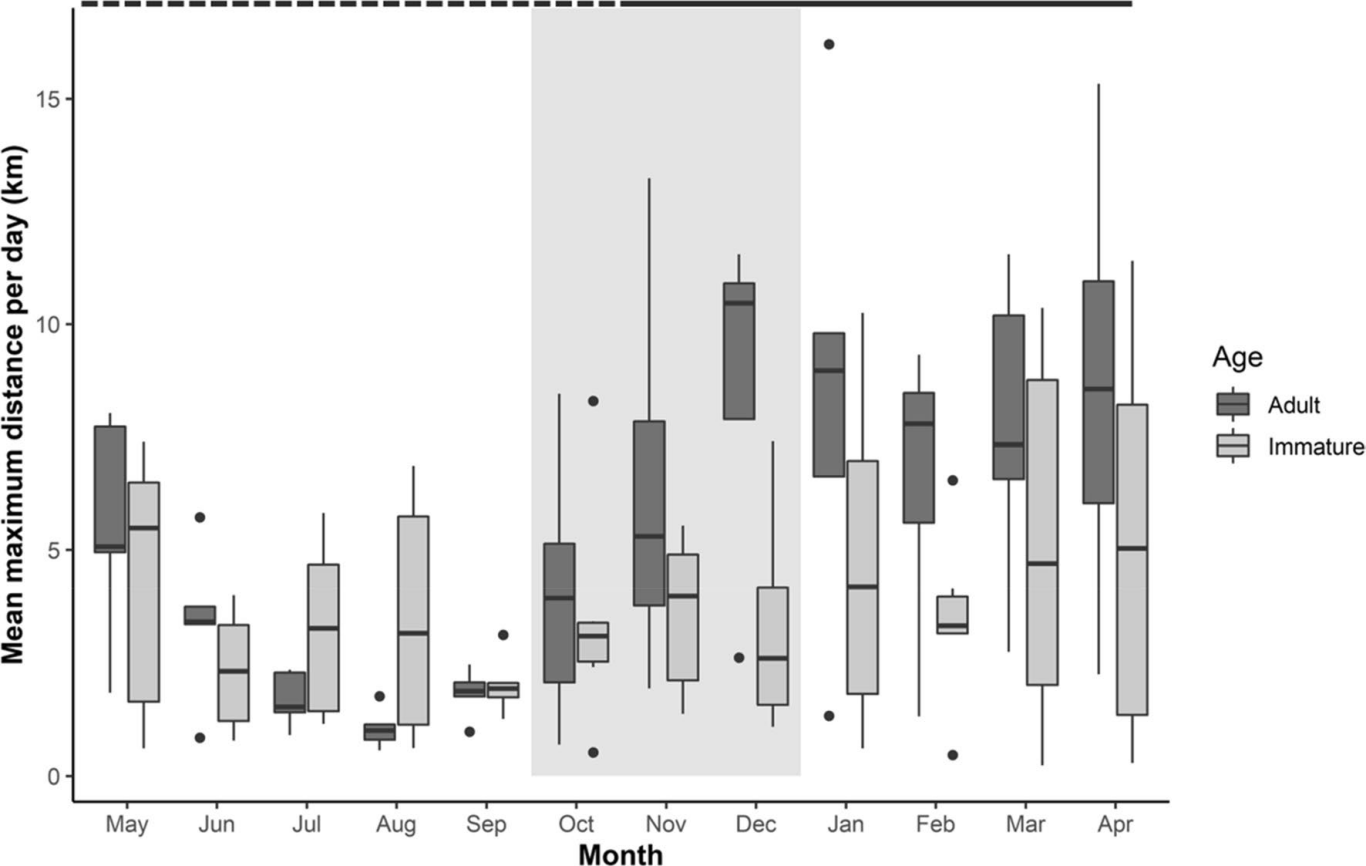
Boxplots of the mean maximum daily distance per month, for individual immature and adult Shoebills, between 2011 and 2018. Data is organised to start at the beginning of the breeding season (May). The boxes represent the 25th, 50th and 75th percentiles of the mean maximum daily distance. Whiskers show 1.5 times the value of inter-quantile range, with values outside this range plotted as black dots. The dashed line above the plot indicates the dry season (May to October) and the wet season (November to April). The shaded area highlights the period between October and December, with an increase of adult mean maximum daily distances.

The mean maximum daily distances moved varied throughout the year, particularly for adult Shoebills. During the breeding season, from June until October, adults performed shorter movements, with the mean maximum daily distance moved being the lowest in August (1.1 km per day). In October, towards the end of the breeding season, adult mean maximum daily distance started to increase, peaking in December (10.5 km per day). Immature Shoebills show less variation in movement distances over the year. Birds moved least in September (1.9 km per day), while movement distances peaked in May to 5.5 km per day (Fig. 3).

### Influence of NDWI on Shoebill movements

On over 80% of the days, Shoebills moved less than 3 km, thus ‘Moving Days’ were defined as days when Shoebills moved more than 3 km (Fig. 2) and the regions where birds stayed for a minimum of two days between Moving Days were classified as ‘Areas’ (further details in the Methods section). Between October and December of 2013–2017, across the five adults we located 39 different Areas, and, in 2014 and 2015, across the 6 immatures, we identified 33 Areas. Immature birds stayed on average 14 ± 19 days in Areas, whereas adult birds spent 17 ± 24 days in Areas before moving to another location. These locations always had negative daily mean NDWI values, indicating that Shoebills were not in open water areas and selected relatively dry regions.

**Figure 4:**
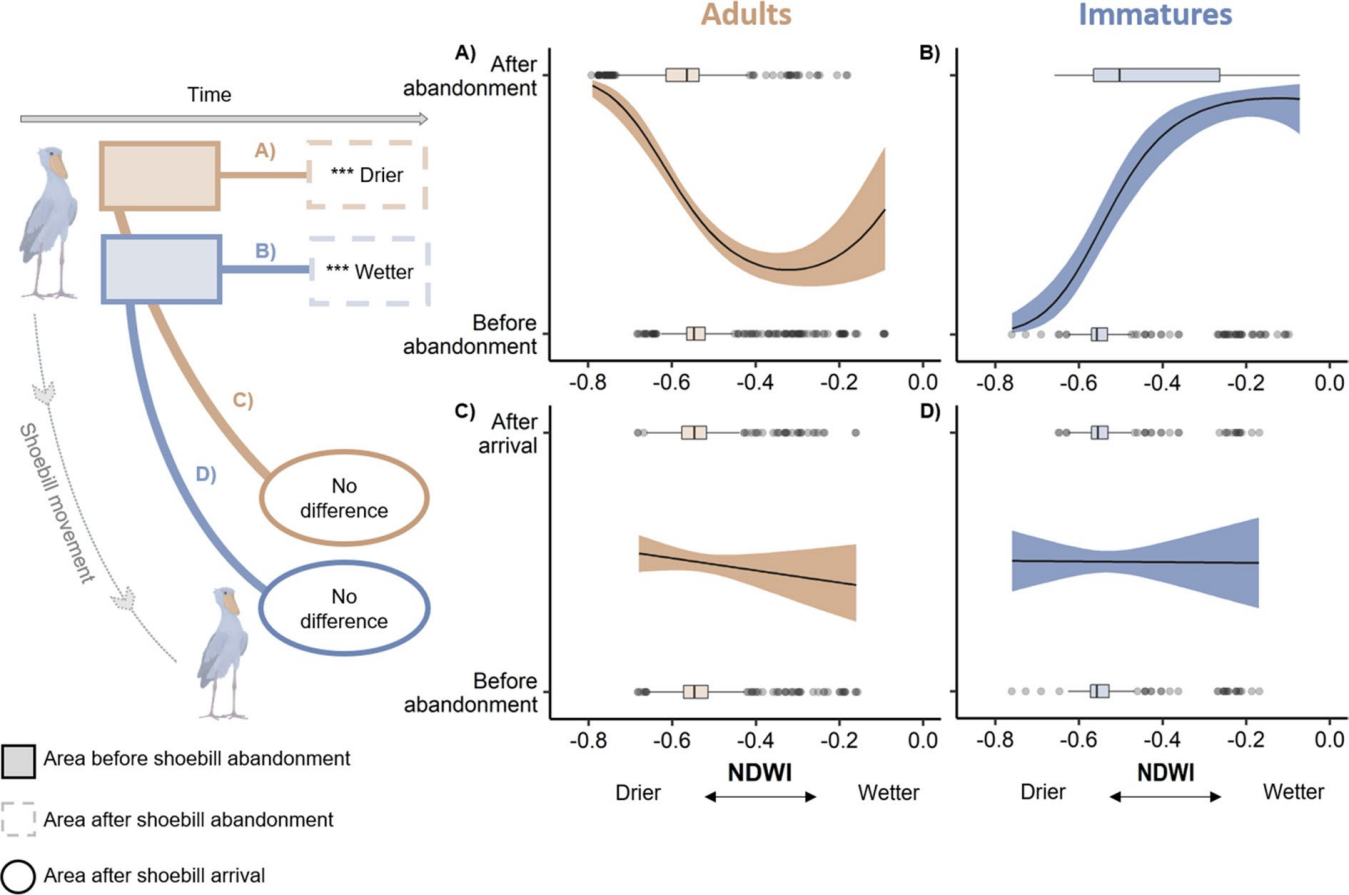
Diagram describing the analysed spatial and temporal relationships of Shoebill movements. The plots show the predicted mean daily NDWI values (solid line) and 95% confidence interval (shaded areas) for **(A)** adults and **(B)** immatures of ‘Areas’ before and after Shoebill abandonment, and for **(C)** adults and **(D)** immatures before Shoebill abandonment and after Shoebill arrival in a new ‘Area’. The boxplots display the observed values of daily mean NDWI, with the boxes representing the 25th, 50th and 75th percentiles and the whiskers the 1.5 times the value of inter-quantile range. Values outside this range are plotted as grey dots. Brown colours indicate adult data and blue colours indicate immature data.

We found that the NDWI of the Areas used by Shoebills between October and December was statistically different from the NDWI of the same Areas the week after the birds abandoned (Table 3), both for immature and adult Shoebills. However, these relationships differed between the age classes. Adult Shoebills used Areas with an average NDWI value of −0.52, varying from −0.68 to −0.10. The week after adults left the Area, it became drier with the NDWI decreasing to an average of −0.57 (range −0.79 to −0.18). In contrast, for immatures, the mean NDWI of Areas used was −0.53, with a minimum NDWI value of −0.76 and maximum of −0.10.After abandonment, the average NDWI of these Areas increased to −0.43 (range −0.66 to −0.07), indicating that the Areas became wetter (Fig. 4A,B). The variance explained by the immature model was higher (marginal R-squared 0.27) than by the adult model (marginal R-squared 0.14), and in both cases the random factors slightly increased the R-squared (immature conditional R-squared 0.32; adult conditional R-squared 0.15) (Table 3).

**Table 3:** Results of the GLMM models comparing the values of: (a) daily mean NDWI of Areas before Shoebill abandonment, with the values of NDWI after the birds abandoned the Area, using year and Area nested within bird ID as random effects. Daily mean NDWI was transformed as a second-degree polynomial (poly 1 and poly 2), (b) daily mean NDWI of used Areas the last week before abandonment, with the daily mean NDWI of used Areas the first week after arrival, using year and Area nested within bird ID as random effects.

Model	Age	Parameter	Estimate (SE)	Z-value	P-value	Marginal R-squared	Conditional R-squared
(a) Comparison of NDWI of areas before and after Shoebill abandonment	Immature	Intercept	0.17 (0.19)	0.87	0.384	0.270	0.320
		NDWI (poly1)	33.87 (3.38)	10.02	< 0.001		
		NDWI (poly2)	−7.35 (2.70)	−2.72	0.007		
	Adult	Intercept	0.06 (0.08)	0.76	0.445	0.144	0.151
		NDWI (poly1)	−23.12 (2.65)	−8.72	< 0.001		
		NDWI (poly2)	15.03 (2.73)	5.50	< 0.001		
(b) Comparison of NDWI of used areas the last week before abandonment and the first week after arrival	Immature	Intercept	−0.03 (0.54)	−0.05	0.963	< 0.001	< 0.001
		NDWI	−0.05 (1.01)	−0.05	0.957		
	Adult	Intercept	−0.50 (0.46)	−1.10	0.272	< 0.001	< 0.001
		NDWI	−0.95 (0.85)	−1.11	0.268

We did not find a statistical difference between the NDWI of Areas used by Shoebills the week before abandonment, and the NDWI of the newly colonised Areas the first week after Shoebill arrival; this was the case for both adults and immatures (Table 3). The mean NDWI of Areas used by adult Shoebills the week before abandonment and the week after arrival was −0.54 (ranging from −0.76 to −0.16). For immatures, the NDWI of Areas before abandonment was −0.49 (varying from −0.76 to −0.07), compared to −0.50 (from −0.66 to −0.09) the week after arrival (Fig. 4C,D).

## Discussion

We described for the first time the annual home range sizes and variation in distances moved over the year for adult and immature Shoebills, providing evidence of age-related differences in their movement ecology. Furthermore, we show that movement patterns of Shoebills were associated with changes in surface water, but these changes contrasted between age classes, with adults abandoning sites that became drier, whereas immatures abandoned sites that became wetter. Despite the small number of tracked Shoebills, which can make the generalisation of our results to other Shoebill populations challenging, this species inhabits similar habitats throughout their narrow distribution range^31^, and thus their movement ecology is likely influenced by analogous environmental drivers.

Shoebills in the Bangweulu Wetlands were largely sedentary, moving less than 3 km on over 80% of days. The main prey of Shoebills in the Bangweulu Wetlands are catfish, which they catch mainly through the tactics of stand and wait on top of floating vegetation^33,34,40^. Indeed, field studies in the Bangweulu Wetlands found that they spent 85% of the time performing low-energy activities, such as standing, sitting and preening^34^. Walking and flying behaviours may also be associated with foraging, given that a Shoebill strike may disturb the prey and require a move to a different location^40^. Therefore, much of these Shoebill’s daily movements were likely related to foraging events or searching for suitable foraging habitat.

The average annual home range of Shoebills was around 1500 km^2^ which is larger than for similar species, such as Abdim’s Storks (*Ciconia abdimii*) in Niger (10–120 km^2^^41^). However, there was large individual variation in home range size, both for adult (304–3375 km^2^) and immature Shoebills (233–2.628 km^2^). Other studies have shown large individual variation in home range size of similar wetland species, such as Wattled Cranes (*Bugeranus carunculatus*), with 95% kernel density estimates varying between 0.4 and 110.4 km^2^^42^, Mauritanian Spoonbills (*Platalea leucorodia balsaci*), with home ranges varying from 23 to 101 km^2^^43^, or American White Pelicans (*Pelecanus erythrorhynchos*) summer home range varying between 177 and 4710 km^2^^44^. These variations in individual home range size in the same habitat and within the same species show that animal movement is more complex than a simple reflection of underlying resource distribution^11,45^, and other factors (e.g. social attraction/repulsion) may also influence individual distribution^46,47^.

Several factors can influence the home range size in birds, such as age^14,15,44,48^, sex^14,44,49^ and degree of individual specialisation in particular foraging areas^50^. Shoebills do not exhibit strong sexual dimorphism, and the birds in this study were not genetically sexed, thus it was not possible to investigate possible sex differences in home range size. We did not find age-related differences in annual home range size, and although 2 individuals slightly decreased their home range size by an average of 173 km^2^ as they aged from immatures to adults, 2 other individuals increased their home range size by an average of 665 km^2^ as they matured. However, there is a suggestion of individual consistency, since the individuals with smallest and largest home ranges as immatures maintained smaller and larger home ranges as adults (Table 2). In many situations, breeding adults have smaller home ranges than non-breeders during part of the year because their movements are constrained by the location of their nest site^15,16^. Although animals in areas of higher productivity tend to have smaller home ranges^11,51^, this might not be the case in swampy areas. In the wet season, with an increase in water levels, Shoebill prey species occupy larger areas of the swamps, forcing birds to increase their home ranges. Adult birds, with more experience, may build up knowledge of the landscape, occupying the most suitable foraging locations and outcompeting less experienced birds^52^. Consequently, a possible increase in adult home range size during the wet period may be counterbalanced by the seasonal constrain of the nest site location, resulting in approximately the same average home range size for adult and immatures.

Indeed, immature Shoebills moved consistent distances throughout the year, while adults moved smaller distances during the breeding season (May–October), particularly during the incubation and chick-rearing period (June–September). During the breeding season, adults forage close to the nest, moving smaller distances and occupying smaller home ranges^15,16^. Shoebills chicks hatch in June-July, and until the chicks are about 40 days old, at least one adult is constantly on the nest^37^. Later in the breeding season (September and October), adult daily distances moved started to increase. Shoebills build their nests on top of floating vegetation^36,53^, but as the breeding season progresses, the water levels recede to the point that by the end of the breeding season, the nests are resting on solid ground^37^. This might also decrease the suitability of the foraging areas surrounding the nest, forcing adult birds to increase their daily moved distances as the breeding season progresses to find suitable foraging sites.

Environmental factors can also determine movement and home range size in birds, and, for water-dependent species, the spatial and temporal distribution of surface water is one of the main drivers of movement^9,10,12^. Bird species respond differently to changes in water availability, with some functional groups responding to sequences of flooding and drying patterns, while others respond immediately to changes in flooded area^54^. For example, Black Storks wintering in West Africa move as the rivers begin to dry^55^, Mallard (*Anas platyrhynchos*) movements are highly predictable and strongly linked to the presence of surface water^10^ and Grey Teal (*Anas gracilis*) fly hundreds of kilometres directly towards temporary water sources^54^. In Southern Africa, the patterns of rainfall and primary productivity are the main drivers of large-scale movements of Egyptian Geese (*Alopochen aegyptiaca*) and Red-billed Teal (*Anas erythrorhyncha*)^12^. Here, we show that drying and flooding patterns of the Bangweulu Wetlands at the start of the rainy season are important drivers in the movement of Shoebills.

In the Bangweulu Wetlands, November marks the start of the rainy season, being the month with lowest water levels in this region^56^. Our results show that between October and December, Shoebills occupied areas of low surface water availability (low NDWI values), which is likely the most available habitat. There were, however, differences in how adult and immature birds responded to changes in surface water. While adults seemed to abandon areas that became drier, immatures abandoned areas that became wetter, suggesting age-related differences in habitat use or foraging strategies. Moreover, the areas selected by Shoebills had the same surface water as the areas they were previously occupying, which suggests a selection for an optimal surface water level by this species. Water-depth limits non-diving waterbirds foraging ranges, by directly restricting the accessibility of the habitats due to birds morphology (e.g. neck and metatarsus)^6^. Consequently, Shoebills foraging locations are also restricted to the water-depths suitable for foraging.

We hypothesise that the different movements in response to surface water between age-classes might be related to prey availability and optimal foraging conditions. Immature birds tend to be less efficient foragers^21,57^ and occupy less optimal foraging locations^52^. Distributions of waterbirds are greatly influenced by the hydrology of the wetlands and distribution of food resources^58^, since different species have different foraging methods and depend on particular water depths and prey vulnerability^6^. Shoebills are typically solitary birds, but they occasionally concentrate in drying pools of water, where fish may become highly abundant^33^. Immature Shoebills may take advantage of this recession of the water level, which promotes the availability of prey^13^ and thus would be suitable areas for immatures to gain experience in capturing prey. Shoebills also forage in deep water, using floating vegetation as fishing sites and then diving forward, described by Guillet^33^ as a “peculiar and complicated technique called collapsing”. Although birds using this technique have lower foraging success than on flooded grassland, the catfish caught in deeper waters are on average larger than on flooded grasslands^34^, as larger catfish prefer deeper waters^34,59^. Therefore, immature birds might prefer drier areas with higher abundance of relatively smaller prey, whereas adults having already mastered the highly specialised deep-water foraging technique, might prefer flooded areas with larger prey, and thus greater rewards per capture. Nonetheless, our interpretations are based on, as yet, unverified validation of the NDWI in swampy areas, particularly the areas used by Shoebills that are typically densely vegetated and have water with low oxygen content^33^, which can pose constraints on the identification of water features using satellite imagery^60^. In future research, newly available satellite imagery (e.g. Sentinel-2, launched in 2015) and recently created indexes (e.g. Xu 2006^60^) may provide further detail on how surface water influences the movements of wetland species. However, these indexes need to be validated in swampy wetlands, which may have their own unique characteristics^61^.

Moreover, changes in water surface may not be the only environmental variable driving the movement of Shoebills. Henry et al*.*^12^ explored the main environmental variables influencing the movement decisions of Egyptian Geese and Red-billed Teal in Southern Africa, and although changes in surface water appeared in several of their models, suggesting that the flooding and drying patterns of wetlands have some predictive power, rainfall and primary productivity were found to be more important in explaining movement patterns in these species. The relatively small variance explained by our models also suggest that other non-measured variables likely play a role in driving the movements of Shoebills; we therefore suggest for future research to complement the use of NDWI with high temporal and spatial records of rainfall and NDVI to further explore the drivers of movement patterns and spatial distribution of Shoebills in the Bangweulu Wetlands.

## Methods

### Study area and data collection

This study was conducted in the Bangweulu Wetlands, a Game Management Area (GMA) located in the Muchinga province in north-eastern Zambia (approximately between 11°40' to 12°34'S and 29°78' to 30°87'E). The Bangweulu Wetlands consist of miombo (*Brachystegia* sp.) woodlands, grasslands, floodplains, seasonal swamps, and permanent wetlands^62^. This reserve is classified as an Important Bird Area and the area of Chikuni is classified as a Ramsar Site^63^. The climate is characterised by a heavy rainfall season from November to April, with a total annual precipitation of 1200 mm to 1400 mm^56,64^. The lowest water levels occurs in November, and the mean annual water level difference is 1.4 m^65,66^. This area harbours the southernmost population of Shoebills^31,38^, however the size of this population is largely unknown. In 1984, the first Shoebill census in the Bangweulu Wetlands estimated the population at 200–300 individuals^67^. Nevertheless, a large area of the wetland remained un-surveyed^62^ and, in a more recent survey, Roxburgh and Buchanan^35^ provided an estimated population size of 1296 individuals, although this estimate was based on very few sightings, and there was considerable uncertainty around this estimate (95% confidence interval: 477–2372).

Between August and September of 2011 to 2014, 10 juvenile and 1 adult Shoebills were fitted with 70 g satellite-based GPS-trackers (Solar Argos/GPS PTTs, Microwave Telemetry) (Table 1). The transmitters were fitted using the body-loop attachment method, with a Teflon-tube harness. Eight pre-fledging juveniles were tagged on their nests when they were on average 84 days old (range 80—89 days). Shoebills fledge at approximately 95–105 days^38^. Two juveniles (511 and 521) were raised in a recovery centre after being confiscated from the illegal bird trade, and fitted with the GPS transmitter before being released at unknown ages, but likely older than 80–89 days of the other birds. Only one Shoebill (517) was tagged as an adult, which was caught at its nest site. Tracking devices, including harness, weighted 80 g, representing 1.3–1.6% of the body mass of birds at the time of deployment (4900–6300 g). Licences to catch and deploy the tracking devices were provided by the Zambia Wildlife Authority (now Department of National Parks and Wildlife (DNPW)), and the work was approved by the University of Cape Town Science Faculty Animal Ethics Committee.

### Data processing and spatial analysis

The trackers provided a GPS fix every 1-h between 6 A.M. and 6 P.M., GMT + 2, which corresponds to the activity period of Shoebills. The transmitters provided location (latitude and longitude) with a mean error of 18 m^68^. We considered all valid GPS locations until the transmitter failed or when there was no movement for several days, indicating death or loss of the GPS transmitter. GPS data was filtered for outliers based on unrealistic movements or speed (more than 150 km/h between two consecutive hourly locations) and visually inspecting the tracks.

Birds were classified as juveniles until the start of the following breeding season (1st of May), as immatures during the second and third year and as adults from the fourth year onwards, since Shoebills start to breed after 3 years^38^. For this study, we only considered the movements of immature and adult birds, since first year juveniles remained near the nest for a long period after fledging^37^. Six individuals provided more than 1 year of data, maturing from juvenile to immature birds, and four immature birds provided more than 3 years of data, becoming adults (Table 1).

We estimated the annual home range area of individual immature and adult birds using Kernel Density Estimation, with *h-ref* algorithm and grid size of 500 m, using R package a*dehabitatHR*^69^. The year was defined from the start of the breeding season (May) until the following April. We also calculated cumulative home ranges of immature and adults, across all years and individuals, to visualise the area used by this species in the Bangweulu Wetlands.

We quantified the maximum range of Shoebill individual daily movements by calculating the distance between all GPS locations each day and selecting the maximum value (hereafter maximum daily distance). All distances were calculated using R package *geosphere*^70^. To understand how movements changed throughout the year for immature and adult birds, we calculated the mean maximum daily distance per month of each individual. All data processing and analysis were performed in R 3.6.1^71^.

### Influence of NDWI on Shoebill movements

We analysed Shoebill movements between 2013 and 2017, in relation to changes in surface water from October to December each year. During this period, the levels of surface water change dramatically in the Bangweulu Wetlands, as the rainy season typically starts in November. This period also encompasses the end of the Shoebill breeding season and birds are less constrained by the location of the nests. We compared the NDWI of areas used by Shoebills prior to and after they abandoned them, and compared the NDWI of used areas the last week before abandonment with the NDWI of the newly selected areas, the first week after arrival.

To understand when Shoebills performed large movements, we analysed the frequency of the maximum daily distances. We defined a size threshold (in km’s) which captured 80% of smaller scale movements and considered the remaining 20% as ‘*Moving Days’*. Here we also accounted for movements performed during the night, by calculating the distance between the first GPS location of the day and the last GPS location of the previous day. Movements performed during the night were allocated to the previous day. We classified as ‘*Areas’*, the regions where birds stayed for a minimum of two days between Moving Days. We computed the 95% minimum convex polygons (MCPs) of these Areas and, to understand if birds moved to a different geographical area or remained in a similar location after a Moving Day, we overlayed the MCPs of two consecutive Areas. If the two MCPs overlapped, we considered the individual to have remained in the same Area; if they did not overlap, we considered that the individual moved to a different Area. MCPs were calculated using R packages *sp*^72,73^ and *adehabitatHR*^69^*.*

**Figure 5:**
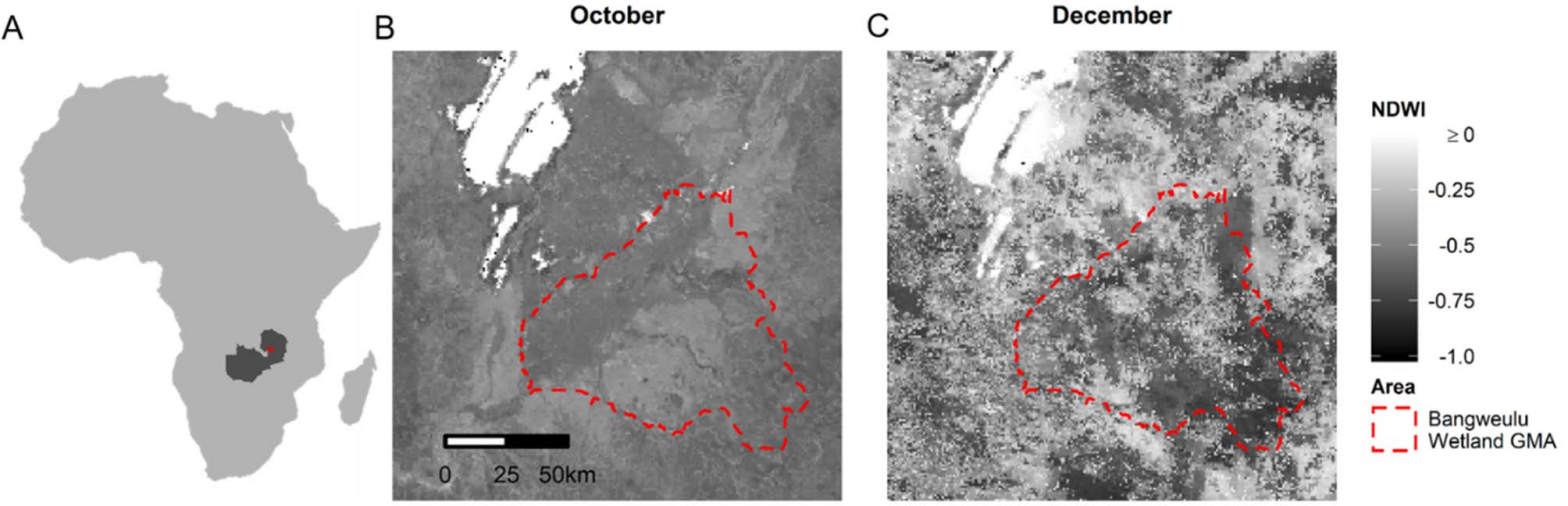
**(A)** Location of the Bangweulu Wetlands Game Management Area (in red) within Africa (light grey) and Zambia (dark grey); **(B)** changes in NDWI between the start of October (week between 30/09/2015 and 07/10/2015), just prior to the start of the rainy season and **(C)** the end of December (week between 19/12/2015 and 26/12/2015), which is during the rainy season. The red dashed line indicates the border of the Bangweulu Wetlands Game Management Area. Satellite imagery obtained from MODIS Terra Surface Reflectance (10.5067/MODIS/MOD09A1.006).

We used the NDWI as a proxy for surface water and calculated this index for the Bangweulu Wetlands for all weeks of October until January. When using satellite imagery there is a trade-off between temporal and spatial resolution. In this study, we favoured imagery with higher temporal resolution, using satellite imagery from MODIS Terra Surface Reflectance with 8-days and 500 m resolution^74^, since the pixel size of 500 m was still smaller than the analysed range of movements. All images had a minimum of 92% of pixels with good quality and a maximum of 2% of pixels not classified due to cloud cover or other reasons. To calculate the NDWI, we used McFeeters^26^ formula:

$$NDWI=\frac{\left(Green-NIR\right)}{(Green+NIR)}$$

where *Green* is MODIS Band 4 (545–565 nm wavelength) and *NIR* (near infrared) is MODIS Band 2 (841–876 nm). The NDWI varies between 1, indicating open water features, and -1, indicating a dry area, on a gradient of surface water. This index was interpreted comparatively, e.g. an area of NDWI of -0.6 is drier than an area of NDWI -0.5 (Fig. 5). All satellite imagery manipulation was performed using R packages *raster*^75^ and *rgdal*^76^ and *rgeos*^77^.

To test if Shoebills move due to changes in surface water, we extracted the daily mean NDWI of the GPS positions of Shoebills while they were in a particular Area. We then compared the locations where birds were present, with the locations one week after the birds abandoned the Area. We used binomials Generalised Linear Mixed Models (binomials GLMMs), with presence (0)/abandonment (1) of Shoebills as the response variable, daily mean NDWI as the fixed effect, and year and Area nested within bird ID as random effects, to account for lack of independence of measures within years and within the Areas used by different Shoebills. Due to the non-linearity of the relationship between Shoebill presence/abandonment and NDWI (as areas Shoebills abandoned could have become drier or wetter, i.e., with lower or larger NDWI values), we introduced the NDWI as a second-degree polynomial term in the GLMM. We calculated the marginal and conditional R-squared, to assess the variance explained by the fixed effect of the model (mean daily NDWI), and the fixed and random effects of the model, respectively. We built two models, one for adults and another for immatures, to evaluate if the two age groups responded differently to changes in surface water.

To understand if Shoebills select areas of similar surface water when they move, we compared the Shoebill locations the first week after arrival (1) with the locations the last week before they abandoned an Area (0). We tested this hypothesis for immatures and adults. We used binomials GLMMs, with newly selected area (1)/previously occupied area (0) as the response variable, mean daily NDWI as a fixed effect, and year and Area nested within bird ID as random factors. We assessed the variance explained by the model using marginal and conditional R-squared. GLMMs were computed using R package *lme4*^78^, and R-squared values computed using the package *MuMIn*^79^.

### Approval for animal experiments

Licences to catch and deploy the tracking devices were provided by the Zambia Wildlife Authority (now Department of National Parks and Wildlife (DNPW)). The work was carried out with approval from the University of Cape Town Science Faculty Animal Ethics Committee (permit number: 2011/V14/AA). Capture, handling and tagging procedures were carried out by RHEM, qualified in 2007 under the Article 9 of the Experiments on Animals Act in The Netherlands. No bird was injured by the capturing/handling procedure.

## Data Availability

The datasets used and analysed during the current study are available from the corresponding author on reasonable request.
